# Association of obesity phenotypes with electrocardiographic subclinical myocardial injury in the general population

**DOI:** 10.1002/clc.23155

**Published:** 2019-02-06

**Authors:** Izzah Vasim, Muhammad I. Ahmad, Morgana Mongraw‐Chaffin, Elsayed Z. Soliman

**Affiliations:** ^1^ Department of Internal Medicine Wake Forest School of Medicine Winston‐Salem North Carolina; ^2^ Department of Internal Medicine, Section on Hospital Medicine Wake Forest School of Medicine Winston‐Salem North Carolina; ^3^ Department of Epidemiology and Prevention Wake Forest School of Medicine Winston‐Salem North Carolina; ^4^ Department of Epidemiology and Prevention Epidemiological Cardiology Research Center (EPICARE), Wake Forest School of Medicine Winston‐Salem North Carolina

**Keywords:** metabolic syndrome, metabolically healthy obesity, NHANES III, subclinical myocardial injury

## Abstract

**Background:**

As the debate continues about whether obesity in metabolically healthy individuals is associated with poor outcomes or not, investigating the association between the obesity phenotypes and markers of subclinical myocardial injury will help identify those at risk for future cardiovascular events (cardiovascular disease [CVD]).

**Hypothesis:**

We hypothesize that obesity phenotypes including metabolically healthy obesity (MHO) is associated with subclinical myocardial injury (SC‐MI).

**Methods:**

This analysis included 3423 participants (57.85 ± 13.06 years, 53.3% women) without known CVD from National Health and Nutrition Examination Survey (NHANES) III. Multivariable logistic regression models were used to examine the cross‐sectional association between four obesity phenotypes (metabolically healthy nonobese (MHNO) [reference], metabolically unhealthy nonobese (MUNO), MHO, and metabolically unhealthy obese (MUO) with SC‐MI. SC‐MI was defined from the 12‐lead electrocardiogram as cardiac infarction/injury score ≥ 10 units. Metabolic syndrome (MetS) was defined according to the International Diabetes Federation consensus definition. Obesity was defined as body mass index ≥30 kg/m^2^.

**Results:**

MUO was associated with higher odds of SC‐MI compared with MHNO (odds ratio [OR], 1.53; 95% confidence interval [CI], 1.22‐1.92, *P* = 0.0005). This association was stronger in men vs women (OR [95% CI]: 2.20 [1.58‐2.07] vs 1.08 [0.79‐1.48]), respectively; interaction *P*‐value = 0.002) but was consistent in subgroups stratified by age and race. There was no significant association of MHO or MUNO with SC‐MI compared with MHNO, but there was a trend toward higher odds of SC‐MI in the MUNO group (*P*‐value for trend across MHNO, MUNO, and MUO = 0.0002).

**Conclusions:**

Our findings suggest that a combination of obesity and MetS confers worse prognosis and early preventive strategies aimed at weight loss and management of MetS components may decrease the risk of future poor outcomes.

## 1 INTRODUCTION

Although metabolic abnormalities and obesity are known risk factors for cardiovascular disease (CVD), a subcategory of obesity without metabolic syndrome (MetS), referred to as metabolically healthy obesity (MHO) has yielded contradictory estimates of association with CVD.^1^, ^2^ MHO was associated with increased CVD risk in previous studies including four meta‐analyses.^3^, ^4^, ^5^, ^6^, ^7^ However, several individual studies did not find any association between MHO and CVD.^8^, ^9^, ^10^

As this controversy about MHO and CVD continues, an examination of MHO and other obesity phenotypes with cardiovascular markers of poor outcomes may provide further evidence of more proximal risks associated with these phenotypes. One such marker is the cardiac infarction/injury score (CIIS), an electrocardiographic‐based scoring system used to define subclinical myocardial injury (SC‐MI).^11^ CIIS has been associated with future adverse events such as coronary heart disease (CHD), cardiovascular and all‐cause mortality.^12^, ^13^, ^14^ An examination of the association of obesity phenotypes with a marker of SC‐MI using 12‐lead electrocardiogram is a simple and cost‐effective way to stratify the CVD risk associated with these phenotypes. Therefore, we sought to examine the cross‐sectional association between obesity phenotypes and SC‐MI in a sample from the third National Health and Nutrition Examination Survey (NHANES III) free of clinically diagnosed CVD. We hypothesized that obesity phenotypes (MUNO, MHO, and MUO) would be associated with prevalent SC‐MI independent of potential confounders.

## 2 METHODS

### 2.1 Study population

NHANES is a periodic survey of a representative sample of the civilian noninstitutionalized US population. Its principal aim is to determine estimates of disease prevalence and health status of the US population. The National Center for Health Statistics of the Center for Disease Control and Prevention Institutional Review Board approved the protocol for NHANES‐III. All participants gave written informed consent. Baseline data were collected during an in‐home interview and a subsequent visit to a mobile examination center between 1988 and 1994. The following characteristics were self‐reported: age, sex, race/ethnicity, income, prevalent CVD, cancer history, smoking status, and leisure time physical activity. Medication history, including the use of antihypertensive agents and lipid‐lowering therapies, also were self‐reported. A physical examination was performed to obtain body mass index (BMI). Blood pressure readings were taken during the in‐home evaluation and again during the mobile examination center visit and averaged for each participant. Fasting blood samples were collected to measure total cholesterol, high‐density lipoprotein cholesterol, triglycerides, and glucose, using laboratory procedures as reported by NCHS.^15^

For this analysis, we only included NHANES‐III participants who underwent an ECG recording (n = 8561). We excluded participants with a history of CVD (myocardial infarction, heart failure, or stroke), any major electrocardiographic abnormalities based on Minnesota Code classification,^16^ taking anti‐arrhythmic drugs, with implanted pacemakers, with cancer on chemotherapy, or missing key covariates. Finally, to be eligible for this analysis, participants must have provided a blood sample after fasting for at least 8 hours. After all exclusions (n = 5138), 3423 participants were included in the final analysis.

Obesity was defined as BMI ≥ 30 kg/m^2^. Obese and nonobese participants were subsequently divided into two subgroups based on the presence of MetS. MetS was defined according to the International Diabetes Federation consensus definition as having three or more metabolic abnormalities^17^ (Supporting information Table S1).

### 2.2 Electrocardiogram

Resting 12‐lead electrocardiograms were obtained with a Marquette MAC 12 system (Marquette Medical Systems, Milwaukee, Wisconsin) during the mobile examination visits by trained technicians. Analysis of electrocardiograms was achieved through a computerized automated process and visual inspection by a trained technician located in a centralized core laboratory. The derivation of the CIIS and methodology have been described previously.^11^ The SC‐MI defined by CIIS is based on a weighted scoring system taking several objective electrocardiographic waveform components related to myocardial injury and ischemia, both discrete and continuous, and generating a risk‐stratified scoring system. This system was designed to improve on previous models that relied on more subjective criteria and decision trees which were vulnerable to the erroneous application at each branch along the decision tree. The score is defined by a combination of 11 discrete and four continuous features and provides a simple scoring scheme suitable for both visual and computer classification of a standard 12‐lead ECG. SC‐MI was defined as CIIS values ≥10 points.^11^, ^12^

### 2.3 Statistical analysis

Baseline characteristics were compared across nonobesity (MHNO and MUNO) and obesity (MHO and MUO). Continuous variables were reported as mean ± SD, while categorical variables were reported as frequency and percentage. Student *t*‐test was used to compare the continuous variables while *χ* ^2^ was used to compare the categorical variables. Multivariable logistic regression analysis was used to compute odds ratios (ORs) and 95% confidence interval (CI) for the cross‐sectional association between each obesity phenotype (MHNO [reference], MUNO, MHO, and MUO) and SC‐MI. We calculated *P* for trend across obesity phenotypes using a multivariable logistic regression model. Two incremental models were constructed: model 1 adjusted for age, sex, race (non‐whites), and socioeconomic status. Model 2 was further adjusted for smoking, physical activity and low‐density lipoprotein (LDL‐C).

We also conducted a subgroup analysis of the association between obesity phenotypes and SC‐MI stratified by age (dichotomized at 65), sex and race (white vs non‐white). The models were adjusted similarly to model 2 as mentioned above.

Additional analyses were performed as follows: First, we performed multivariable linear regression analysis with each obesity phenotype (MHNO [reference] MUNO, MHO, and MUO) as the independent variable and CIIS as the continuous outcome variable to calculate the adjusted mean ± SE. Models were adjusted as mentioned above. Secondly, to assess whether increasing BMI is associated with higher CIIS, we examined the association of BMI categories (18.5‐24.9 (reference), 25‐29.9, 30‐34.9, 35‐39.9, and ≥ 40) with CIIS using linear regression models. Model 1 was adjusted for age, sex, non‐whites, socioeconomic status and model 2 adjusted for model 1 plus smoking, physical activity, LDL‐C, and all MetS components.

All statistical analyses were performed using with SAS version 9.4 (SAS Institute Inc, Cary, NC), and *P*‐values were considered significant if less than 0.05.

## 3 RESULTS

This analysis included 3423 participants (57.85 ± 13.06 years, 53.3% women, 49.3% non‐Hispanic whites) of whom 49.6%, 21.8%, 9.6%, and 18.8% had MHNO, MUNO, MHO, and MUO, respectively. SC‐MI was present in 21.7% (n = 744) of participants. The prevalence of SC‐MI was 19.3% in MHNO, 24.3% in MUNO, 16.9% in MHO and 27.4% in MUO participants. Table 1 shows baseline characteristics of participants stratified by nonobesity and obesity.

**Table 1:** Baseline Characteristics of the study participants

Characteristics, mean ± SD or n (%)	Nonobesity		*P*‐value^a^	Obesity		*P*‐value^a^
	Metabolically healthy n = 1700	Metabolically unhealthy n = 748		Metabolically healthy n = 331	Metabolically unhealthy n = 644	
Age (years)	57.2 ± 13.3	62.0 ± 13.1	<0.0001	52.8 ± 11.2	57.1 ± 11.6	<0.0001
Male (%)	853 (50.1%)	362 (48.4%)	0.41	110 (33.2%)	271 (42.0%)	0.007
Non‐Hispanic white	883 (51.9%)	408 (54.5%)	0.23	114 (34.4%)	285 (44.2%)	0.003
Total annual family income <20 000	678 (40.3%)	346 (47.2%)	0.001	146 (44.9%)	330 (52.4%)	0.02
Systolic blood pressure (mm Hg)	125.51 ± 18.20	137.38 ± 18.54	<0.0001	125.20 ± 16.49	135.72 ± 16.93	<0.0001
Diastolic blood pressure (mm Hg)	74.14 ± 9.56	77.93 ± 10.25	<0.0001	76.69 ± 8.90	79.24 ± 9.93	<0.0001
Insulin resistance (%)	438 (25.8%)	543 (72.6%)	<0.0001	61 (18.6%)	458 (71.1%)	<0.0001
Triglycerides (mg/dL)	111.0 ± 64.1	203.6 ± 117.2	<0.0001	113.9 ± 74.1	207.8 ± 192.5	<0.0001
HDL cholesterol (mg/dL)	57.3 ± 16.8	44.1 ± 13.3	<0.0001	54.1 ± 12.8	43.9 ± 12.0	<0.0001
LDL‐C (mg/dL)	132.4 ± 38.1	142.0 ± 37.1	<0.0001	136.4 ± 35.8	142.3 ± 41.1	0.02
Waist circumference (cm)	88.0 ± 9.1	97.2 ± 12.8	<0.0001	106.3 ± 10.4	110.7 ± 10.0	<0.0001
Body mass index (Kg/m^2^)	24.2 ± 2.9	26.6 ± 2.4	<0.0001	33.5 ± 3.6	34.3 ± 4.4	0.005
Smoking (%)						
Current smoker	457 (26.8%)	172 (22.9%)	0.04	54 (16.3%)	120 (18.6%)	0.37
Former smoker	534 (31.4%)	246 (32.8%)	0.47	82 (24.7%)	223 (34.6%)	0.001
Never smoker	709 (41.7%)	330 (44.1%)	0.26	195 (58.9%)	301 (46.7%)	0.0003
Physical activity (METs per week)^b^	13.6 (2.3‐34.8)	10.1 (1.0‐31.3)	0.001	7.0 (0.8‐24.4)	5.8 (0‐24.4)	0.36
SC‐MI (%)	329 (19.3%)	182 (24.3%)	0.005	56 (16.9%)	177 (27.4%)	0.0002
Cardiac infarction/injury score	4.8 ± 6.4	5.6 ± 7.1	0.009	4.2 ± 5.7	6.1 ± 7.4	<0.0001

Abbreviations: LDL‐C, low‐density lipoprotein cholesterol; HDL, high‐density cholesterol; SC‐MI, subclinical myocardial injury; METs, metabolic equivalent.

a *P*‐value by student *t* test for continuous and *χ* ^2^ for categorical variables.

b METs reported as median and IQR.

Among obesity group, MUO were more likely to be old, men, white, smokers, and to have low annual income and physical activity levels compared to MHO. Among the nonobesity group, MUNO were more likely to be old, women, white, nonsmokers, and to have low annual income and physical activity levels compared to MHNO.

In multivariable models adjusted for potential confounders, MUO was associated with higher odds of SC‐MI (OR 1.53; 95% CI, 1.22‐1.92, *P* = 0.0005). There was no statistically significant association between MHO or MUNO with SC‐MI; However, a trend of higher odds of SC‐MI was observed in MUNO (*P*‐value for trend across MHNO, MUNO, and MUO = 0.0002) (Table 2). A similar pattern of association was observed when using CIIS as a continuous variable, as shown in Table 3, there was a pattern of higher mean values in MUO followed by MUNO in multivariable linear regression models.

**Table 2:** Multivariable odds ratio and 95% CI of association between obesity phenotypes and subclinical myocardial injury

Obesity phenotypes	Model 1^a^		Model 2^b^	
	Odds ratio (95% CI)	*P*‐value	Odds ratio (95% CI)	*P*‐value
Healthy nonobese	Reference		Reference	
Unhealthy nonobese	1.20 (0.97, 1.49)	0.72	1.14 (0.92, 1.42)	0.95
Healthy obese	0.97 (0.70, 1.34)	0.11	0.96 (0.69, 1.33)	0.16
Unhealthy obese	1.60 (1.29, 1.99)	0.0001	1.53 (1.22, 1.92)	0.0005

Abbreviations: LDL‐C, low density‐lipoprotein cholesterol.

a Model 1 adjusted for age, sex, non‐whites and socioeconomic status.

b Model 2 adjusted for model 1 plus smoking and physical activity and LDL‐C.

**Table 3:** Least mean square and SE of cardiac infarction/injury score across obesity phenotypes

Obesity phenotypes	Model 1^a^	Model 2^b^
	Mean ± SE	Mean ± SE
Healthy nonobese	4.92 ± 0.16	4.98 **±** 0.16
Unhealthy nonobese	5.27 ± 0.24	5.11 ± 0.25
Healthy obese	4.78 ± 0.37	4.76 ± 0.38
Unhealthy obese	6.28 ± 0.26	6.20 ± 0.27

Abbreviation: LDL‐C, low‐density lipoprotein cholesterol.

Least square mean and SE calculated from multivariable linear regression.

a Model 1 adjusted for age, sex, non‐whites and socioeconomic status.

b Model 2 adjusted for model 1 plus smoking and physical activity and LDL‐C.

In subgroup analysis, heterogeneity in the association between obesity phenotypes and SC‐MI was observed by sex. With MHNO as a reference, all obesity phenotypes and particularly MUO had a stronger association with SC‐MI in men compared to women (OR [95% CI]: 2.20 [1.58‐2.07] vs 1.08 [0.79‐1.48] respectively; interaction *P*‐value = 0.002). There was no statistically significant interaction by age or race (Table 4).

**Table 4:** Multivariable odds ratios and 95% CI for the association between obesity phenotypes and subclinical myocardial injury in subgroups

Subgroups	SC‐MI, n (%)	Obesity phenotype	Odds ratio (95% CI)	Interaction *P*‐value
Male	99/362 (27.3%)	Unhealthy nonobese	1.52 (1.12‐2.07)	0.002
	24/110 (21.8%)	Healthy obese	1.36 (0.82‐2.25)	
	89/271 (32%)	Unhealthy obese	2.20 (1.58‐3.07)	
Female	83/386 (21.5%)	Unhealthy nonobese	0.83 (0.60‐1.14)	
	32/221 (14.4%)	Healthy obese	0.71 (0.46‐1.10)	
	88/373 (23.5%)	Unhealthy obese	1.08 (0.79‐1.48)	
White	109/408 (26.7%)	Unhealthy nonobese	1.11 (0.83‐1.48)	0.69
	17/114 (14.9%)	Healthy obese	0.74 (0.42‐1.29)	
	83/285 (29.1%)	Unhealthy obese	1.46 (1.06‐2.01)	
Non‐white	73/340 (21.4%)	Unhealthy nonobese	1.19 (0.85‐1.67)	
	39/217 (17.9%)	Healthy obese	1.13 (0.75‐1.72)	
	94/359 (26.1%)	Unhealthy obese	1.62 (1.17‐2.24)	
Age > 65 y	86/295 (29.1%)	Unhealthy nonobese	1.05 (0.75‐1.47)	0.59
	12/53 (22.6%)	Healthy obese	0.83 (0.42‐1.65)	
	48/159 (30.1%)	Unhealthy obese	1.20 (0.79‐1.81)	
Age ≤ 65 y	96/453 (21.1%)	Unhealthy nonobese	1.26 (0.94‐1.68)	
	44/278 (15.8%)	Healthy obese	0.96 (0.66‐1.40)	
	129/485 (26.6%)	Unhealthy obese	1.70 (1.30‐2.23)	

Abbreviation: LDL‐C, low‐density lipoprotein cholesterol.

Reference group = metabolically healthy nonobese.

Model adjusted for Age, sex, non‐whites, socioeconomic status, smoking and physical activity and LDL‐C.

**Figure 1:**
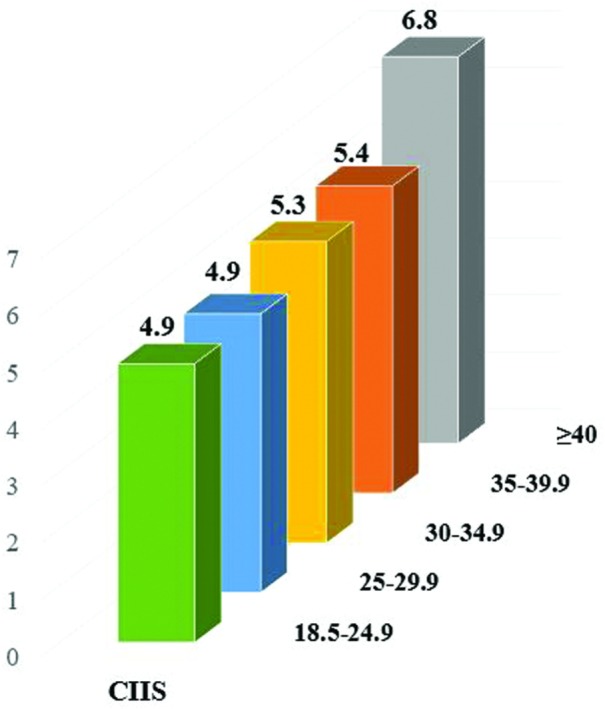
Mean cardiac infarction/injury score (CIIS) across body mass index (BMI) categories. Mean CIIS across BMI categories in a model adjusted for age, sex, race, socioeconomic status, smoking, physical activity, low‐density lipoprotein cholesterol (LDL‐C), insulin resistance, hypertension, elevated triglyceride (TG), Low high‐density lipoprotein (HDL), and high waist circumference (WC)

Table S2 and Figure 1 show the results of linear association of BMI with CIIS. Higher values of CIIS score were observed with increasing BMI independent of socio‐demographic and CVD risk factors (trend *P*‐value 0.22).

## 4 DISCUSSION

We examined the cross‐sectional association between obesity phenotypes and SC‐MI using data from NHANES III. The key findings of our study are as follows^1^: The prevalence of SC‐MI was highest in MUO followed by MUNO, MHO, and then MHNO^2^; Among obesity phenotypes, MUO was significantly associated with higher odds of SC‐MI, and there was trend towards more abnormalities in MUNO following MUO^3^; A similar pattern of higher mean values of CIIS in MUO followed by MUNO was observed^4^; heterogeneity in association between obesity phenotypes and SC‐MI by sex was observed where the association was stronger in men vs women^5^; there was an incremental increase in CIIS with higher values among those participants with higher BMI independent of MetS.

These results taken together indicate that the combination of obesity and MetS is associated with higher odds of SC‐MI and mean values of CIIS. BMI and other measures of adiposity have been consistently associated with CVD, and the major impact of obesity on cardiovascular health is mediated by accompanying metabolic abnormalities.^18^ Observation of higher CIIS with increasing BMI also suggests that excess weight is not without consequences and thus challenges the notion that obesity can be healthy.

MHO is considered a transient state; the duration and severity of obesity leads to an unhealthy state with the passage of time, thus increasing the risk for CVD events.^6^, ^19^ Therefore, a cross‐sectional examination may limit the adequate assessment of potential risks associated with MHO. This may explain the weaker association between MHO with SC‐MI which did not reach statistical significance when compared to MHNO.

We observed a strong association of obesity phenotypes with SC‐MI among men. Men generally have a higher incidence of CHD and higher age‐adjusted CHD mortality rate compared to women.^20^, ^21^, ^22^ Our findings of gender differences in the association of SC‐MI add to accumulating evidence of sex/gender differences in the prevalence and outcomes of different CVD. Future investigation should assess whether genetic background, emerging risk factors, access to health care, awareness, and adherence of medications contribute to sex differences.

In previous studies, MUNO had similar CVD risks as those with MUO.^3^, ^4^ In support of these findings, we observed higher odds of SC‐MI with MUNO, especially in men, suggesting that maintaining metabolic health remains important even in the absence of obesity. Finally, the MUO group has consistently exhibited an unfavorable prognosis in terms of CVD events and mortality across all studies,^3^, ^4^ likely due to the cumulative effect of obesity and MetS. A strong association of MUO with SC‐MI in our study supports these findings.

Our study has certain limitations. First, we are unable to establish a temporal relationship between obesity phenotypes and SC‐MI due to the cross‐sectional design of the study; However, it is unlikely that SC‐MI leads to obesity and Mets, but the opposite is more plausible. Some of the measurements such as smoking and physical activity are self‐reported and thus subject to recall bias. Finally, we adjusted for several confounders, but residual confounding remains a possibility. Strengths of the study include a large sample size and a community‐living multiracial population with generalizability to the US population, as well as the fact that the key variables were ascertained using standard protocols.

## 5 CONCLUSION

Our results in NHANES‐III provide evidence that MetS may contribute to myocardial injury especially in those with obesity. Also, the higher the obesity class based on BMI, the higher is the risk of myocardial injury independent of MetS. With the increasing prevalence of obesity and MetS, it is important to identify high‐risk populations so that finite resources can be allocated to appropriate groups.

## CONFLICTS OF INTEREST

The authors have no conflicts of interest to report.

## Supporting information


**Table S1**. Definition of metabolic syndrome and obesity phenotypes.
**Table S2**. Association of cardiac infarction/injury score (CIIS) with body mass index (BMI) categories.
