# Supplementary material for: Association of obesity phenotypes with electrocardiographic subclinical myocardial injury in the general population
Source: Clin Cardiol. 2019 Feb 6;42(3):373–8. doi: 10.1002/clc.23155 (PMC6712312; doi:10.1002/clc.23155)
Supplement: Supplementary file 1 — Table S1. Definition of metabolic syndrome and obesity phenotypes. Table S2. Association of cardiac infarction/injury score (CIIS) with body mass index (BMI) categories. [file CLC-42-373-s001.docx]

**Online Supplementary Materials**

**Association of Obesity Phenotypes with Electrocardiographic Subclinical Myocardial Injury in the General Population**

**Table S1:** Definition of Metabolic Syndrome and Obesity Phenotypes

**Table S2:** Association of CIIS with BMI Categories

| **Table S1**. **Definition of Metabolic Syndrome and Obesity Phenotypes** |
| --- |
| **Harmonized International Diabetes Federation criteria for MetS: ≥3 of the following components:** |
| TG level ≥150mg/dL or drug treatment for elevated TG. |
| HDL cholesterol <40mg/dL in men and <50mg/dL in women or drug treatment for low HDL cholesterol. |
| Systolic blood pressure ≥130mmHG or diastolic blood pressure ≥85mmHG or blood pressure medications |
| Fasting glucose ≥100 mg/dL or medications for diabetes |
| Waist circumference of ≥102 cm in men and ≥88 cm in women |
| **Obesity Phenotypes** |
| MHNO: BMI <30 kg/m^2^ without MetS |
| MUNO: BMI <30 kg/m^2^ with MetS |
| MHO: BMI>30 kg/m^2^ without MetS |
| MUO: BMI>30 kg/m^2^ with MetS |
| BMI, body mass index; TG, Triglycerides; SBP, systolic blood pressure; DBP, diastolic blood pressure; HDL, high-density lipoprotein; MHNO, metabolically healthy non-obese; MUNO, metabolically unhealthy non-obese; MHO, metabolically healthy obese; MUO, metabolically unhealthy obese |

**Table S2. Association of CIIS with BMI Categories**

| BMI Categories  Kg/m^2^ | Model 1  Beta-Coefficient (95% CI) | p-value | Model 2  Beta-Coefficient (95% CI) | p-value |
| --- | --- | --- | --- | --- |
| 18.5-24.9 | *Ref* | *-* | *Ref* | *-* |
| 25-29.9 | 0.23 (-0.30, 0.76) | 0.39 | -0.01 (-0.71, 0.68) | 0.97 |
| 30-34.9 | 0.77 (0.13, 1.42) | 0.01 | 0.33 (-0.65, 1.32) | 0.51 |
| 35-39.9 | 1.37 (0.37, 2.36) | 0.007 | 0.56 (-0.82, 1.96) | 0.42 |
| ≥40 | 1.37 (-0.15, 2.89) | 0.07 | 1.93 (-0.008, 3.88) | 0.05 |

Model 1 adjusted for age, sex, non-whites and socioeconomic status

Model 2 adjusted for smoking, physical activity, LDL-C, insulin resistance, hypertension, elevated TG, Low HDL and elevated WC

BMI, body mass index; CIIS, cardiac infarction/injury score, LDL-C, low density lipoprotein cholesterol; TG, triglycerides; HDL, high-density lipoprotein; WC, waist circumference
